# Effects of Calculus in the Female Child

**Published:** 1841-11

**Authors:** George A. Rees


*Effects of Calculus in the Female Child.* By George A. Rees, M. R. C. S.—The following is the only case of the kind I have met with in the female out of nineteen thousand children who have been under my care; I consider, therefore, that if briefly recorded it might be worthy of notice in your valuable Journal.

Ruth Mole, aged four years, was brought to me laboring under retention of urine, the mother stating that the child had not passed any water for two days and nights, and that the bowels had not acted during the same time.

July 12. There is considerable fever; great pain; constant moaning; the head hot, and tossed from side to side; the pulse small and frequent; the tongue dry, and covered with a brownish coating; there is some delirium; the abdomen is hot and tense; the bladder perceived to be much distended, extending up to the umbilicus; the external organs of generation are inflamed; the clitoris distended; the nymphæ slightly œdematous.

The distress of the child demanding immediate relief, a flexible catheter was introduced, and twelve ounces of turbid urine were drawn off, and an active aperient was ordered.

13. Immediate relief followed the abstraction of the urine, and the child slept for four hours. The bowels have acted twice freely; there is constant inclination to go to stool, and considerable straining, causing the bowel to prolapse. No water has passed since yesterday; the bladder is again palpably distended, and the same state of the external organs perceptible, but the fever is much abated.

The prolapsus ani and the state of the external organs of generation so analogous to what occurs in boys with retention of urine from urethral calculus (in whom erection of the penis with œdema of its integuments are the principal symptoms,) led to the suspicion that the cause of retention in this instance might be calculus, which suspicion was found to be correct by the introduction of a probe into the urethra. It was, therefore, determined to leave the bladder as it was, unless urgent symptoms supervened, in the hope that the pressure of the urine might expel the stone from the passage.

14. The child is much the same in all respects, but the urine has dribbled away in small quantities since yesterday. The stone may be felt with a probe still lodging in the urethra. After a little trouble this was caught hold of by means of a small pair of common forceps, and brought forward to the orificium urethræ, through which its size prevented its coming without violence sufficient to produce laceration; a small incision was, therefore, made, as less likely to be followed by incontinence of urine, and the stone extracted.

16. All symptoms relieved, but there is incontinence of urine.

22. The child is free from all symptoms, the incontinence of urine having ceased for the last four days.

The calculus is five lines in diameter, weighs eleven grains, and is nearly perfectly round. I believe a calculus of any other shape *could* hardly produce such symptoms in the female child.—*Lancet; Med. and Surg. Jour.*

